# Individual differences in spatial working memory strategies differentially reflected in the engagement of control and default brain networks

**DOI:** 10.1101/2023.07.07.548112

**Published:** 2023-08-22

**Authors:** Nina Purg, Masih Rahmati, Youngsun T. Cho, Anka Slana Ozimič, Aleksij Kraljič, John D. Murray, Alan Anticevic, Grega Repovš

**Affiliations:** a Department of Psychology, University of Ljubljana, Ljubljana, Slovenia; b Department of Psychiatry, Yale University School of Medicine, New Haven, CT, USA; c Department of Psychology, Yale University School of Medicine, New Haven, CT, USA; d Department of Physics, Yale University, New Haven, CT, USA

**Keywords:** spatial memory, fMRI, fine-grained representation, categorical representation, cognitive strategy

## Abstract

Spatial locations can be encoded and maintained in working memory using high-precision, fine-grained representations that are cognitively demanding, or coarse and less demanding categorical representations. In this study, we employed an individual differences approach to identify brain activity correlates of the use of fine-grained and categorical representations in spatial working memory. We combined data from six fMRI studies, resulting in a sample of 153 (77 women, 25 ± 6 years) healthy participants performing a spatial working memory task. Our results showed that individual differences in the use of spatial representations in working memory were associated with distinct patterns of brain activation, with fine-grained representations requiring greater engagement of attentional and control brain systems, while categorical representations were associated with decreased inhibition of the default network. These findings may indicate a greater need for ongoing maintenance and protection against interference for fine-grained compared to categorical representations.

## Introduction

Working memory (WM), the ability to encode and maintain information in support of an ongoing task, plays a critical role in many complex cognitive processes such as learning, reasoning, problem solving, and language (D’Esposito and Postle, 2015). To support the wide range of cognitive processes and abilities, information stored in WM can be encoded and maintained using a variety of representations and strategies. The specific representations and strategies used depend on several factors, such as the type of information to be retained (Oblak et al., 2022; Slana Ozimič et al., 2023; Starc et al., 2017), the response to be generated (Purg et al., 2022), the predictability of the response (Curtis, 2004; Purg et al., 2022) and the available attention resources (Adam et al., 2015; Starc et al., 2017). Increasingly, research also shows that individuals, even when faced with the same task requirements, may use different representations and strategies to perform the task (e.g., Oblak et al., 2022; Slana Ozimič et al., 2023; Starc et al., 2017). Here, we investigate neural correlates of individual differences in WM strategy use in a large multi-study, multi-site dataset of spatial WM performance during functional magnetic resonance imaging (fMRI).

Spatial working memory (sWM) enables the maintenance and manipulation of encoded spatial information (e.g., location of an object). Animal neurophysiology studies have related sWM performance to persistent activity in the frontal and parietal brain areas, which is thought to reflect active encoding and maintenance processes (Chafee and Goldman-Rakic, 1998; Funahashi et al., 1989; Fuster, 1973; Fuster and Alexander, 1971; Kubota and Niki, 1971). The directional selectivity of persistently activated neurons has suggested that the information maintained may represent the direction towards the target stimulus location or a motor response plan (Funahashi et al., 1993; Medendorp et al., 2005; Takeda, 2004; Zhang and Barash, 2000, 2004). In humans, fMRI studies have similarly identified WM-related persistent activity in a number of brain regions, ranging from the frontal and parietal cortices to the posterior sensory areas (Brown et al., 2004; Courtney et al., 1998; Curtis, 2004; Srimal and Curtis, 2008; Zarahn et al., 1999). However, the specific information represented by this activity has been more difficult to identify. Relating brain activity with behavioral performance measures, studies have shown that brain activity varies with the level of response precision (Curtis, 2004; Hallenbeck et al., 2021), specific strategy use (Curtis, 2004; Purg et al., 2022), general memory load (Adam et al., 2018; Glahn et al., 2002; Leung et al., 2004; Linden et al., 2003; Proskovec et al., 2019) and behavioral prioritization (Klyszejko et al., 2014; Yoo et al., 2022).

The variety of information reflected in persistent activity related to sWM performance is consistent with the observation that individuals report the use of different representations and strategies to encode and maintain spatial information in WM (e.g., Oblak et al., 2022; Slana Ozimič et al., 2023; Starc et al., 2017). In our recent work (Purg et al., 2022), we have shown that the type of representation used in sWM depends on the characteristics and demands of the task. We biased participants towards the use of retrospective sensory or prospective motor coding by changing the predictability of response in different task conditions, which was reflected in distinct patterns of brain activation and functional connectivity. However, there is a high variability in the representations and strategies used even when participants are faced with the same task requirements (Oblak et al., 2022; Slana Ozimič et al., 2023; Starc et al., 2017). This variability can be observed from trial to trial during sWM performance of the same individual due to internal factors, such as attentional fluctuations (Klyszejko et al., 2014; Starc et al., 2017; Yoo et al., 2022). For example, previous studies (Huttenlocher et al., 2004, 1991; Purg et al., 2022; Starc et al., 2017) have shown that participants can encode the stimulus location in WM in terms of precise fine-grained information that requires stronger engagement of attentional resources or as a coarse categorical representation that is cognitively less demanding, such as storing spatial information in the form of a limited number of spatial categories (e.g., “up”, “down”, “left”, and “right”). These representations can be used simultaneously during a sWM task, and their use can be dissociated based on their effect on behavioral responses. Specifically, the use of categorical representations results in a systematic shift of the average response towards the categorical center, while the use of the fine-grained representation is reflected in the response precision estimated as the variability around the average response (Huttenlocher et al., 2004, 1991).

In our previous work (Starc et al., 2017), we separately estimated the degree of reliance on fine-grained and categorical representations in the performance of a sWM task, while measuring pupil responses. We assumed that increased pupil dilation would reflect increased cognitive effort exerted toward the formation and maintenance of either fine-grained or categorical representations. Our results showed a compensatory use of fine-grained and categorical spatial coding from trial to trial within individuals. A drop in attentional resources directed towards the formation of precise fine-grained representations during the encoding of a target location resulted in increased reliance on categorical representations during the late maintenance and response phases of the task. Similarly, in a separate fMRI study (Anticevic et al., 2010), we have investigated the relationship between the response accuracy in a WM task and brain activity during the task. Our results showed that stronger deactivation in the temporo-parietal junction (TPJ) and the default network during the encoding of stimulus location predicted a higher accuracy of WM performance. Since these brain regions have been associated with stronger deactivation during increased cognitive effort and suppression of distractors (Raichle, 2015a; Shulman et al., 2003; Todd et al., 2005), these results suggest that increased attentional demands are required for the formation of detailed representations and protection against interference.

Although both studies (Anticevic et al., 2010; Starc et al., 2017) focused on within-subject variability in behavioral responses, Starc et al. (2017) have also shown a variability across participants in their tendency to use fine-grained or categorical spatial representations. Specifically, we observed that individuals who showed on average worse response precision also exhibited greater categorical bias, reflecting overall higher reliance on categorical representations. This observation suggests that the use of specific representations and strategies might reflect stable individual differences. However, to the best of our knowledge, no studies have yet explored the neural correlates of individual differences in the use of sWM strategies that affect response precision. Previous studies have primarily focused on the neural correlates of WM capacity (e.g., Adam et al., 2015; Dong et al., 2015; Liu et al., 2018; Luck and Vogel, 2013; Minamoto et al., 2015; Osaka et al., 2003; Xu et al., 2018). These studies have shown that individual differences in WM capacity correlate with the level of brain activation (e.g., Adam et al., 2015; Dong et al., 2015; Liu et al., 2018; Luck and Vogel, 2013; Minamoto et al., 2015; Osaka et al., 2003; Xu et al., 2018) and are often attributed to the level of attentional engagement exerted towards the maintenance of information in WM (Adam et al., 2015; Astle and Scerif, 2011; Chow and Conway, 2015; Unsworth et al., 2014; Unsworth and Robison, 2015). On the basis of these findings, we explored whether attentional mechanisms might also affect the use of fine-grained and categorical representations.

**Fig. 1.**
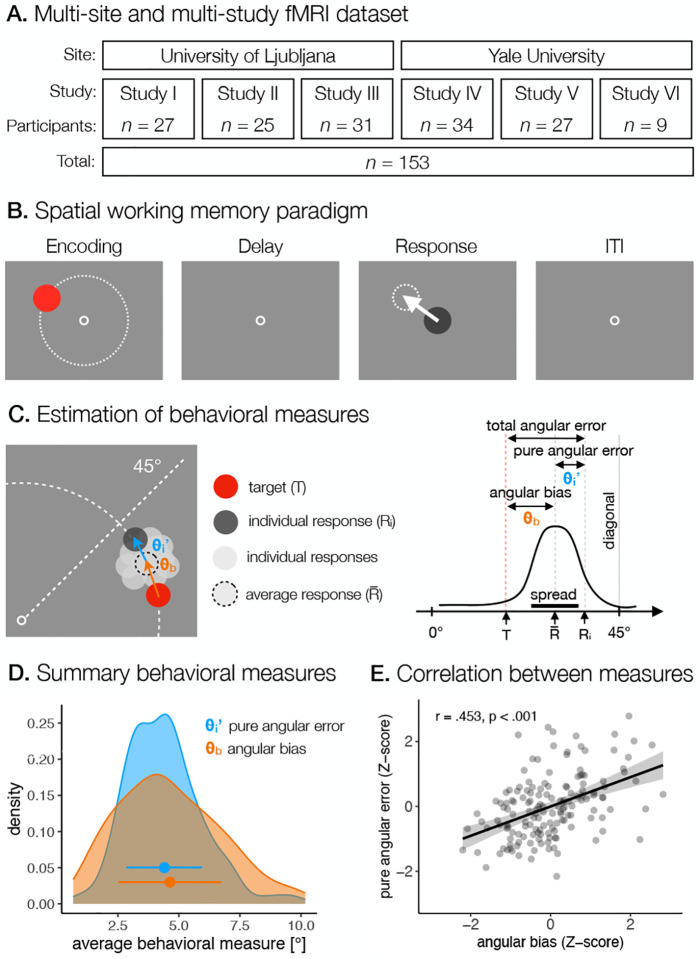
Behavioral paradigm and performance. **A.** The dataset included six fMRI studies related to sWM, conducted at two different sites. In total, 153 participants were included in the data analysis. **B.** Common elements of sWM tasks in all studies. Each task trial consisted of a brief presentation of a target stimulus at different angles and a constant amplitude from the center of the screen, followed by a hand response to the target location using a joystick after a short delay. ITI refers to the inter-trial interval. **C.** An illustration of how the pure angular error (*θ′*) and the angular bias ($\theta_{b}^{'}$) were calculated. **D.** The distribution of pure angular error and angular bias across participants, respectively. The points present the mean of each measure, with the range presenting the standard deviation of the measure. **E.** Pearson’s correlation between pure angular error and angular bias across all participants.

In this study, we were interested in brain activity correlates of individual differences in the use of fine-grained and categorical spatial coding in WM. Due to the hypothesized relationship between the use of these WM strategies and the level of cognitive effort required, we focused on brain systems that have previously been related to general engagement of attention and cognitive control, specifically the dorsal attention, the frontoparietal, and the cingulo-opercular networks, and the default network (e.g., Barch et al., 2013; Cole et al., 2014; Ji et al., 2019; Raichle, 2015a; Smith et al., 2009). We hypothesized that a stronger reliance on precise fine-grained representations would be supported by increased activation of attentional and control systems, and a stronger inhibition of the default network, whereas the reliance on categorical information would be related to decreased engagement of attentional and control systems, and decreased suppression of the default network. To test this hypothesis, we used response precision and categorical response bias as proxies for the use of fine-grained and categorical representations, respectively, and tested their association with brain responses measured with fMRI during a sWM task (Figure 1B). A methodological challenge in the investigation of individual differences in brain-behavior relationships are low effect sizes that require large sample sizes to be detected (Elliott et al., 2020; Grady et al., 2021; Marek et al., 2022). To overcome this flaw, we combined six different sWM fMRI studies conducted at two different recording sites (Figure 1A). Together, we used data from 153 (77 women, 25 ± 6 years) healthy individuals, which largely exceeded average sample sizes in similar studies (e.g., around 25 participants, Marek et al. (2022)). Our study revealed individual differences in the use of spatial representations in WM that were related to distinct patterns of brain activation, with fine-grained representations associated with stronger engagement of attentional and control brain systems, while categorical representations were linked to decreased inhibition of the default network. These findings suggest that fine-grained representations may pose a greater cognitive demand for ongoing maintenance and protection from interference compared to categorical representations.

## Materials and Methods

### Participants

We combined data from six studies (Figure 1A). Three studies (I-III; Table S1) were conducted at the University of Ljubljana, Slovenia, and three studies (IV-VI; Table S1) at Yale University, USA. Between 11 and 37 participants took part in each study, for a total of 166 participants. All participants were healthy adults without current or previous neurological, psychiatric, or substance-use disorders. The exclusion criteria also included any contraindications to MR, such as the presence of metal implants or any other metal particles in the body, a history of epileptic seizures, tremor or other motor disorders, and pregnancy. All participants had normal or corrected-to-normal vision. Several participants were excluded from further data analysis due to incomplete data collection (*N* = 4), failure to follow instructions (*N* = 1), poor data quality or excessive movement during data collection (*N* = 2), and errors during data processing (*N* = 1). Furthermore, we excluded any outliers in data (*N* = 5), which is explained in detail in sections Behavioral data analysis and *fMRI preprocessing and analysis*. Data from the remaining 153 (77 women, 25 ± 6 years) participants were used for further analysis. Most of the participants were right handed (92.1%), while the rest of the participants were left-handed (5.92%) or ambidextrous (1.97%). All participants performed the behavioral task with their dominant hand. Detailed demographic information of participants included in the data analysis is presented in Table S1. The studies carried out at the University of Ljubljana were approved by the Ethics Committee of the Faculty of Arts, University of Ljubljana, and the National Medical Ethics Committee, Ministry of Health of the Republic of Slovenia. Studies conducted at Yale University were approved by the Yale Institutional Review Board. Participants gave written informed consent before participating in the study. In all studies, participants had to perform a sWM task while their brain activity was measured with fMRI.

### Spatial working memory (sWM) task

Behavioral tasks slightly differed between individual studies, therefore, we analyzed only those task conditions that were directly comparable across studies. In particular, we focused our investigation on the task condition in which participants were asked to remember the position of a briefly presented target stimulus and, after a delay period, to move a probe using a joystick to the position of the remembered target (Figure 1B). Despite minor differences in task design, the goal of the task was always the same and there were no differences in task difficulty. The task was displayed on an MR-compatible screen, which was visible to participants from the MR scanner via a head mirror. Specific screen sizes and resolutions varied between recording sites and studies, which is described in detail in Table S2. The tasks were prepared using custom scripts and run in PsychoPy (Studies I–III; Table S2) (Peirce et al., 2019) or E-Prime 2.0 (Studies IV-VI; Table 2) (Schneider et al., 2012). Participants gave their responses to the task using an MR-compatible joystick (Hybridmojo LLC, Washington, USA).

The sWM task differed in the time course of task events and the exact range of target locations across studies (for details see Table S2). In three studies (I-III; Table S2), the trial started with the presentation of a fixation point (2.5 s) in the center of the screen, followed by a brief presentation of a target disk stimulus. In the remaining three studies (IV-VI; Table S2), the trial started immediately with the presentation of a target disk stimulus. The target presentation lasted between 0.1 s and 2 s, depending on the study. The target stimuli were presented at variable locations that were pseudorandomly chosen from 20 to 36 different possible locations, depending on the study. Target locations were chosen so that the target amplitude from the center of the screen was constant for each participant, while the target angles from the center of the screen varied between trials for the same participant (see Table S2 for details on the exact target amplitudes and angles for each study). Target stimuli were never presented on the cardinal axes to prevent verbalization of precise locations (Srimal and Curtis, 2008). Participants were instructed to remember the exact position of the target stimulus. In one study (I; Table S2), the target presentation was followed by a masking pattern (0.05 s) with the aim of disrupting iconic visual memory (Curtis, 2004). In all studies, the target presentation was followed by a delay period (8 s to 10.4 s, depending on the study) during which a fixation point was presented in the center of the screen, instructing the participants to fix their gaze in the center of the screen. In three studies (IV-VI; Table S2), gaze-fixation was additionally enforced by instructing participants to push a button upon change of color of the fixation cross, which occurred randomly in 50% of all trials. After the delay, the probe appeared (a disk stimulus of the same size as the target stimulus, but of different color) in the center of the screen, and participants were asked to move the probe using a joystick to the location of the previously presented target stimulus, as precisely as possible. The time of their response was limited due to the concurrent fMRI recording between 2.3 s and 3 s, depending on the study. Individual trials were separated by an inter-trial interval (ITI), which was either fixed in duration (Studies IV-VI; Table S2) or randomly varied in duration to allow better task-related BOLD signal decomposition (Studies I-III; Table S2). Participants performed between 20 to 80 trials of the task, split between 1 to 4 blocks, depending on the study.

### Behavioral data analysis

In behavioral data analysis, we first converted all behavioral data from pixel-based measurements into degrees of visual angle (°va) to provide standardization across different screen resolutions and viewing distances. At the level of individual participants, we calculated trial-to-trial response errors, which are thought to reflect the precision of the sWM. We used the final location of the response in relation to the target location to assess the precision of the behavioral responses. Since the findings of single-neuron recordings suggest that spatial representations at the neuronal level are encoded in terms of angle and amplitude in the polar coordinate system (e.g. Chafee and Goldman-Rakic, 1998; Funahashi et al., 1989; Rainer et al., 1998), we decomposed the response error on each trial into angular and amplitude differences between target and response locations measured from the center of the screen (Figure S1A).

Next, we excluded any invalid or outlier responses to ensure that the results reflected the engagement of sWM and not any technical errors or inattention to the task. We employed several criteria to identify outliers based on individual task trials. First, any response that was located more than 45° from the target location in either direction or whose amplitude was not between 0.5 or 1.75× the target amplitude was excluded from further analysis. Due to the potentially large impact of outliers, we additionally excluded any responses that fell outside of 1.5× IQR limits for either an angular or an amplitude error. In total, we excluded on average 8.42% of trials per participant.

Several studies (Haun et al., 2005; Huttenlocher et al., 2004, 1991; Purg et al., 2022; Starc et al., 2017) have suggested the presence of systematic effects on behavioral responses, such as systematic under- or overshooting of responses and the tendency to shift responses closer to the nearest diagonals. To estimate any systematic biases in behavioral responses, we used a modified procedure described in Starc et al. (2017). Specifically, we calculated the systematic amplitude bias as an average amplitude error, with negative values reflecting an undershoot and positive values reflecting an overshoot. We calculated the systematic angular bias as an average angular error at each distinct angle from the nearest diagonal, with positive values reflecting response bias towards the diagonal and negative values reflecting response bias away from the diagonal. We estimated systematic biases for each participant separately and then subtracted them from the original recorded trial-to-trial responses to obtain pure amplitude error and pure angular error.

To obtain an overall measure of the precision of the response, we computed the average pure angular error for each individual. For an overall measure of systematic angular bias, we calculated the root-mean-squared-difference (RMSD) across bias estimates at distinct target angles for each participant. Based on the overall performance, we identified one outlier participant that exceeded the threshold of 3*xSD* based on the overall measure of systematic angular bias. To account for the potential influence of different study designs and protocols on behavioral performance, we standardized behavioral measures for participants within each study before combining data across studies for further analyses. We computed correlation between behavioral measures across participants using the Pearson’s correlation co-efficient.

### fMRI acquisition, preprocessing and analysis

fMRI data were collected with Philips Achieva 3TX, Siemens Tim Trio, and Prisma scanners. We acquired T1- and T2-weighted structural images and several BOLD images using T2*-weighted echo-planar imaging sequences. We also collected pairs of spin-echo images with opposite phase encoding to estimate field maps for the purpose of distortion correction during data preprocessing. The acquisition parameters for specific images varied between different studies, as described in Table S3.

Preprocessing and analyses of MRI data were performed using Quantitative Neuroimaging Environment and Toolbox (QuNex) (Ji et al., 2023). Several steps of analysis and visualizations were prepared using R (Team, 2022a), Matlab (R2021a, Natick, Massachusetts, USA), and Connectome Workbench (Human Connectome Project, Washington University, St. Louis, Missouri, USA).

MR images were preprocessed using Human Connectome Project (HCP) minimal preprocessing pipeline (Glasser et al., 2013). Specifically, structural images were corrected for magnetic field distortions and registered to the MNI atlas, brain tissue was segmented into white and gray matter, and the cortical surface was reconstructed. Functional BOLD images were sliced-time aligned, corrected for spatial distortion, motion-corrected, registered to the MNI atlas and the BOLD signal was mapped to the joint surface volume (CIFTI) representation and spatially smoothed (*σ* = 4 mm). Further analyses were performed on “dense” whole-brain data (i.e. each grayordinate independently). To observe general patterns across functional brain systems and to increase statistical power, we also performed analyses on parcellated whole-brain data. Parcellated data were obtained by extracting the mean signal from specific cortical parcels as identified in the HCP-MMP1.0 parcellation (Glasser et al., 2016) and, in addition, for subcortical parcels and networks based on Cole-Anticevic Network Partition (Ji et al., 2019).

We performed activation analysis using a general linear modeling (GLM) approach in which event regressors were convolved with the assumed double-gamma hemodynamic response function (HRF) Friston et al. (1998). For each participant, we separately modeled each phase of a task trial. Specifically, we estimated the *β* coefficients for the encoding, delay, and response phases (Figure S1B). For three studies (IV-VI; Table S2), we also separately modeled the attention cue in the middle of the delay period using unassumed modeling (Figure S1B). Trials with outlier responses based on behavioral data analysis were modeled as separate events using unassumed modeling and excluded from the group-level statistical analyses of the fMRI data. We additionally modeled motion parameters, their first derivatives, and squared motion parameters to account for any signal artifacts due to movement. To identify outlier participants based on brain activity, we computed Pearson’s correlations and RMSD between delay-related *β* maps across participants. We identified four participants who deviated more than 3*xSD* from the group average *β* map and excluded these participants from further analysis.

Next, to identify significant activation and deactivation during the task, we analyzed *β* estimates at the group level using permutation analysis (500 permutations, tail acceleration) in PALM (Winkler et al., 2014). Specifically, to test the significance of *β* estimates based on the “dense” grayordinate data, we conducted two-tailed one-sample t-tests with TFCE (*H* = 2, *E* = 0.5, *C* = 26) FWE correction. To test the significance of *β* estimates based on parcellated data, we conducted two-tailed one-sample t-tests with FDR correction. The resulting corrected *p*-value maps were thresholded at the whole-brain corrected significance level of *α* < 0.05.

### The estimation of brain-behavior relationship

To estimate the relationship between brain activity in specific networks and behavioral measures, we performed linear modeling with factors pure angular error and angular bias, while study was modeled as a random effect, for each task phase separately. Linear modeling was performed using the lme4 package (Bates et al., 2015) in R (Team, 2022a). To obtain standardized *β* coefficients, brain activity estimates, pure angular error, and angular bias were standardized to *μ* = 0, *σ* = 1 across participants within each study. Correction for multiple comparisons across networks was made using FDR and significant results were identified at *α* < 0.05. For the purpose of investigating the effect of sample size on the detection of brain-behavior relationships, we performed linear modeling on sample sizes from 15 to 153 participants. At each sample size, 1000 samples were created by sampling with replacements from the set of all participants. We then performed linear modeling of the delay-related activity using pure angular error and angular bias as fixed effects in the model and study as a random effect for each separate sample.

To estimate the posterior probability of positive or negative brain-behavior relationship, we specified a Bayesian two-level normal linear model with factors pure angular error and angular bias. We used the study number as the grouping variable on the first level to model varying intercepts across studies. Weakly informative prior distributions were used for all model parameters. Specifically, we used normal prior distributions (*μ* = 0, *σ* = 10) for regression parameters and half-Cauchy prior distributions (*μ* = 0, λ = 2.5) for standard deviations, as recommended by Gelman (2006). The stability of the Markov chain Monte Carlo (MCMC) sampling algorithm was analyzed by verifying that all estimated parameters had estimated effective sample sizes in the bulk of distributions and in the tails of the distributions larger than 400 samples (Vehtari et al., 2021) and that the potential scale reduction statistics ($\hat{R}$) did not deviate from 1.0. To ensure a stable convergence of our models, we visually inspected the trace plots of the posterior parameters and performed prior and posterior predictive checks. We checked that the maximum tree depth was not saturated. Strong degeneracies inherent to multilevel models were addressed by reparametrizing the models to a non-centered parameterization (Betancourt and Girolami, 2013). The models in this study were numerically estimated using the probabilistic programming language Stan (Team, 2022b). The effects of sample size were obtained in the same manner as described in the previous paragraph.

## Results

### Individual differences in the use of spatial coding strategies

We first focused on behavioral indicators of the use of categorical representations during performance of the sWM task (Figures 1C ans S1A). Investigating the response error patterns revealed that participants systematically biased their response toward the nearest diagonal, with a larger bias occurring for the target angles further from the nearest diagonals (Figures S2A and S2C). This finding indicates the use of categorical spatial coding, where participants formed spatial categories defined by the four quadrants of the screen, delineated by the vertical and horizontal axes, each best represented by its central value, i.e., the diagonal (Huttenlocher et al., 2004, 1991; Starc et al., 2017).

Next, to separately measure the contribution of precise and categorical representations in sWM, we decomposed the total response error into two components, pure angular error and angular bias (Figure 1C). We used the pure angular error as a measure of the quality of fine-grained representations, and the angular bias as a measure of the extent to which participants relied on categorical representations. For each behavioral measure, we calculated a summary estimate for each participant, reflecting their use of precise and categorical representations (for distributions across participants, see Figure 1D). For the purpose of combining data from different studies to increase sample size and statistical power, we checked for any discrepancies in behavioral measures between studies using one-way ANOVA (Figures S2B–C). We found differences between studies for both, pure angular error (*F* (5,147) = 8.57, *p* < 0.001, $\eta_{G}^{2}=0.226$) and angular bias (*F* (5,147) = 7.55, *p* < 0.001, $\eta_{G}^{2}=0.204$). To ensure comparibility of results between studies and to prevent the influence of systematic differences, we standardized values for participants within each study before performing analyses on the combined data.

We then investigated the relationship between both measures across all studies, which we estimated using the Pearson’s correlation coefficient. Our results revealed a positive correlation between pure angular error and angular bias (Figure 1E), suggesting that participants who relied more heavily on categorical representations showed poorer precision of fine-grained representations and vice versa.

### Task-related brain activity across different levels of parcellation

In the analysis of the fMRI data, we first investigated the areas of the brain that were activated or deactivated during different phases of a task trial, namely the encoding, delay, and response phases (Figure S3A). During all phases of the trial, significant activation (i.e., *p* < 0.05 corrected for multiple comparisons) was observed in a number of brain regions, extending across the frontal, parietal, and occipital cortices. Subcortical activation was consistently observed in the cerebellum, thalamus, putamen, caudate, and brainstem. Phase-specifc activations differed mainly in the early and ventral visual stream areas, where extensive activation was observed only during the encoding and response phases. Significant deactivation was observed in all phases of the trial in the posterior cingulate cortex, and within areas of the medial prefrontal cortex, and inferior frontal cortex. Additional deactivation was observed in the lateral temporal cortex for delay and response, and in the inferior parietal cortex, early and ventral stream visual areas for the delay phase only. Subcortical deactivation was observed mainly during the delay and response phases in the cerebellum, hippocampus, and amygdala.

To investigate the integration of activity within functional parcels and networks, and their average responses to the task, we also performed activation analysis on fMRI data averaged within cortical parcels of the HCP-MMP1.0 parcellation (Glasser et al., 2016), and within subcortical parcels and networks based on the Cole-Anticevic Network Partition (Ji et al., 2019). Results based on parcellated data showed additional significant task-related activations and deactivations (Figures S3B, S3C and 2A). Focusing on more general networks, increased activity was observed during encoding in the primary and secondary visual networks, the somatomotor, cingulo-opercular, dorsal attention, frontoparietal, and language networks, in addition to the posterior and ventral multimodal networks. Deactivation was observed only in the default network. The delay phase showed significant activation in secondary visual, somatomotor, cingulo-opercular, dorsal-attention, and posterior multimodal networks. In contrast, decreased activity was observed in the default, ventral multimodal, and orbito-affective networks during the delay. Lastly, the response phase was characterized by activation in the primary and secondary visual networks, somatomotor, cingulo-opercular, dorsal attention, frontoparietal, auditory, posterior multimodal, and ventral multimodal networks. A significant deactivation was again observed only in the default network.

Lastly, we checked whether the analysis on parcellated fMRI data improved effect sizes or, alternatively, diluted effects due to inhomogeneous activity within individual parcels and networks. Specifically, similar to the analysis described in Glasser et al. (2016) and Ji et al. (2019), we compared the unthresholded Z-values for delay-related activity between the “dense” grayordinate and parcellated data, and additionally between the parcellated and network data (Figure S3D). Our results showed that although the Z-values of the individual grayordinates and parcels exceeded the Z-values obtained for the parcels and networks to which they belonged, the analysis of the parcellated data resulted in higher overall effect size estimates than the analysis of the grayordinate data. Similarly, analysis of network average data resulted in higher effect size estimates than analysis of the parcellated data. Although working with grayordinate or parcellated data provides better spatial precision of results and is preferable when precise localization is of interest, these results suggest that working with parcellated or network data is preferable when testing hypotheses related to functional regions or networks, as was the case in our study.

### Individual differences in spatial coding strategies reflected in brain activity

**Fig. 2.**
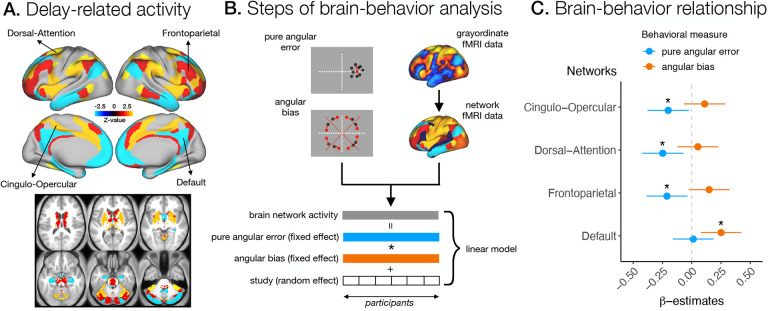
Average network activity in relation to individual spatial working memory performance. **A.** Delay-related activity in cingulo-opercular, frontoparietal, dorsal-attention, and default networks. **B.** Steps in the analysis of the relationship between brain activity in specific networks and behavioral measures of pure angular error and angular bias. For each participant, we computed the average brain activity within networks of interest defined by Cole-Anticevic Network Partition (Ji et al., 2019), and summary measures of pure angular error and angular bias. Next, we ran a hierarchical linear model across participants predicting brain network activity with pure angular error and angular bias, and controlling for study as a random effect. **C.** The relationship between the delay-related activity of specific networks and the behavioral measures of pure angular error (blue) and angular bias (orange). Points indicate *β*-estimates of the main effects, and lines a 95% confidence interval. The asterisks (*) indicate relationships with FDR-corrected *p* < 0.05.

Next, we were interested in whether individual differences in the use of fine-grained and categorical representations are reflected in brain activity. To this end, we used linear modeling to predict activity of brain networks of interest from summary measures of pure angular error and angular bias (Figure 2B). Specifically, we used hierarchical linear modeling with behavioral measures and their interaction as fixed factors and study as a random effect. We focused on the average activity within networks (i) to identify the engagement of broad brain systems during the use of different spatial coding strategies and (ii) to increase the effect sizes and statistical power of the analysis. Our hypothesis was that the use of working memory strategies would be reflected in the engagement of general attention and control brain systems. Specifically, we expected that the use of fine-grained representation would be reflected in increased delay activity in three task-positive networks that are consistently activated in a variety of cognitive tasks, cingulo-opercular, dorsal-attention, and frontoparietal networks (e.g. Barch et al., 2013; Cole et al., 2014; Fox et al., 2005; Smith et al., 2009). In contrast, we hypothesized that reduced cognitive engagement during the employment of robust categorical representation would be reflected in increased activation in the task-negative default network, which consistently shows deactivation during the performance of cognitive tasks in a wide range of studies (for a review, see Raichle (2015a)).

Our results did not reveal significant relationships between brain activity and behavioral measures during the encoding phase in any of the networks of interest (Figure S8A). However, we observed significant brain-behavior relationships during the delay and response phases (i.e., *p* < 0.05 corrected with FDR, Figure 2C and S8A). Specifically, we identified a negative relationship between delay activity and pure angular error for cingulo-opercular (*β* = −0.201, *f*^2^ = 0.028, *p* = 0.036), dorsal-attention (*β* = −0.248, *f*^2^ = 0.049, *p* = 0.025), and frontoparietal networks (*β* = −0.211, *f*^2^ = 0.030, *p* = 0.036), suggesting that increased fine-grained precision was related to increased activity in these networks during sWM performance (Figure 2C). On the other hand, we observed a positive relationship between delay activity and angular bias for the default network (*β* = 0.251, *f*^2^ = 0.058, *p* = 0.019), showing that weaker deactivation in this network was associated with increased use of categorical representations in sWM (Figure 2C). We also observed a negative relationship between brain activity during the response and the pure angular error for cingulo-opercular (*β* = 0.213, *f*^2^ = 0.044, *p* = 0.049) and dorsal-attention networks (*β* = −0.204, *f*^2^ = 0.037, *p* = 0.049) (Figure S8A). The general whole-brain patterns of the relationship between brain activity and both behavioral measures based on “dense” grayordinate, parcellated, or network fMRI data can be observed in Figures S5–7.

We have extended the focused network analysis by computing posterior probabilities of a positive or negative relationship between behavioral measures and brain network activities within a Bayesian statistics framework. The results supported statistical testing and indicated a 98.6%, 99.3%, and 98.8% posterior probability of a negative relationship between pure angular error and delay activity in cingulo-opercular, dorsal-attention, and frontoparietal networks, respectively. The results also indicated a 99.5% probability of a positive relationship between angular bias and default network activity during the delay phase of the task (see Figure S8B for details). The posterior probabilities of a negative relationship between pure angular error and brain activity in the cingulo-opercular and dorsal attention networks during response were 98.8% and 98.5%, respectively (Figure S8B). We also observed a 95.4% posterior probability of a negative relationship between encoding activity in the frontoparietal network and angular bias (Figure S8B).

### The effect of sample size on the detection of brain-behavior relationships

A comparatively large multi-study sample provided us with an increased power to detect brain-behavior relationships with relatively small effect sizes. To further validate the stability of the results and assess statistical power in evaluating brain-behavior relationships, we performed a comprehensive resampling analysis. Specifically, for each sample size from 15 to 153, we randomly selected a set of subjects from our original sample with replacement 1000 times and repeated the hierarchical linear regression for the four networks of interest for the delay period for each sample. This allowed us to evaluate the effects of sample size on model estimates, their confidence intervals, effect sizes, and statistical power.

**Fig. 3.**
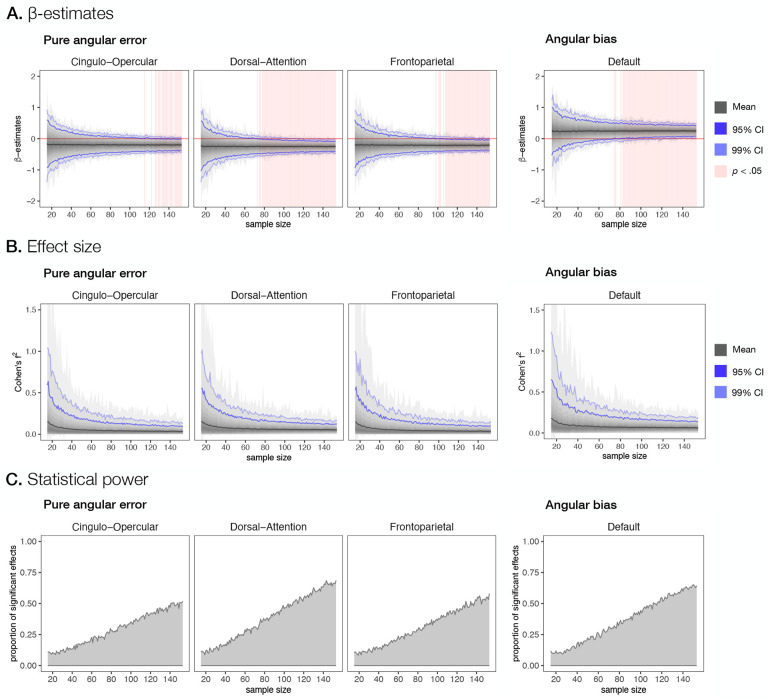
The effect of sample size on the ability to detect significant brain-behavior relationships. We investigated the effect of sample size on **A.** *β*-estimates, **B.** effect sizes and **C.** statistical power in the investigation of the relationship between brain activity and behavioral measures of pure angular error and angular bias. Pure angular error and angular bias estimates were regressed on delay-related activity for each participant in sample sizes from 15 to 153 participants using a hierarchical model with study as a random effect variable. At each sample size, 1000 samples were created, each by sampling with replacements from the set of all participants. **A-B.** Black line denotes mean across all samples, grayed area denotes the span between maximum and minimum value with darker shading reflecting higher density. The lighter blue line denotes the upper and lower boundary for 99% of the samples, the darker blue denotes the upper and lower boundary for 95% of the samples, **A.** Red line denotes 0 value, pink background shading denotes sample sizes for which the 95% confidence interval does not include 0. **C.** The proportion of samples with statistically significant effects is shown. Significance was evaluated at *α* < 0.05 with FDR correction for multiple comparisons.

Whereas mean *β*-coefficients estimated in the linear model were generally stable across different sample sizes (Figure 3A), our results indicated that the variability of *β*-estimates across samples within each sample size changed significantly with sample size. Zero was robustly excluded from the 95% confidence interval computed across 1000 re-samplings for the significant relationships observed in our study only after sample sizes of 146, 78, 108, and 83 participants for relationships with cingulo-opercular, dorsal-attention, frontoparietal, and default networks, respectively. The effect sizes computed as Cohen’s *f*^2^ values decreased exponentially with increasing sample size (Figure 3B). In addition, statistical power, computed as a proportion of FDR-corrected significant effects within a sample size, linearly increased with increasing sample size and reached 51.9%, 68.7%, 58.1%, and 64.3% for relationships with cingulo-opercular, dorsal-attention, frontoparietal, and default networks, respectively (Figure 3C).

## Discussion

A spatial location can be encoded and maintained in WM using different representations and strategies. Fine-grained representations provide detailed stimulus information but are cognitively demanding. On the other hand, categorical representations may be a less demanding strategy, but at the cost of lower response precision. In our study, we were interested in the extent to which individuals rely on fine-grained and categorical representations to encode and maintain spatial information in WM and how these individual differences in sWM strategies are reflected in brain activity.

### Individual differences in spatial coding strategies

Our results showed the presence of a categorical bias in the behavioral performance of the sWM task. Specifically, we observed a robust systematic angular bias, showing that participants tended to move their responses closer to the nearest diagonals. Several previous studies (Haun et al., 2005; Huttenlocher et al., 2004, 1991; Purg et al., 2022; Starc et al., 2017) have suggested that such bias reflects the use of categorical representations – participants spontaneously impose spatial categories in coding stimulus position, which are defined as four quadrants that are delineated by the horizontal and vertical axes. Similar findings were obtained in a series of experiments by Huttenlocher et al. (1991), where participants had to remember and reproduce the location of a dot within an empty circle with responses consistently moved towards the diagonals of four quadrants. This bias was replicated even when the experimenters imposed different spatial categories by clustering stimuli around the horizontal and vertical axes, as well as encouraging participants to use categories centered on the cardinal axes and bounded by the diagonals (Huttenlocher et al., 2004). This suggests that the horizontal and vertical axes present the most exact category boundaries, resulting in the lowest misclassification of spatial information (Huttenlocher et al., 2004).

Huttenlocher et al. (2004, 1991) have argued that the use of categorical and fine-grained representations is not mutually exclusive – individuals may encode both fine-grained and categorical representations of a stimulus location. While the fine-grained representation contains information about the precise location of the target, it can still be affected by a loss of precision or inexactness, leading to a spread of responses around the precise position (Huttenlocher et al., 1991). Based on individual sWM performance, we obtained separate estimates of the degree of reliance on categorical representations and the loss of precision of fine-grained representations for each participant. We were particularly interested in the relationship between the use of these types of spatial coding strategies. Our results replicated previous observation of a positive correlation between loss of fine-grained memory precision and the use of categorical coding Starc et al. (2017). Specifically, our results suggest that there are individual differences in the balance between the use of fine-grained and categorical coding – individuals with higher fine-grained precision of spatial representations relied less on categorical information, whereas individuals who showed lower precision in fine-grained representations seemed to rely more strongly on categorical representations.

Fine-grained and categorical representations appear to have compensatory roles, where uncertainty or loss of fine-grained memory precision is compensated by increased reliance on coarse categorical representations (Huttenlocher et al., 1991; Starc et al., 2017). Similarly, in an earlier study (Starc et al., 2017) we have reported a compensatory use of fine-grained and categorical representations on a trial-by-trial basis within the same individual, where the failure to encode fine-grained information with high precision at the time of encoding of spatial information could then be compensated for using categorical information in the late delay and response periods of the trial. At the interindividual level, the degree of reliance on categorical versus fine-grained representations has been related to individual working memory capacity (Crawford et al., 2016; Stukken et al., 2016). Studies of working memory capacity have traditionally focused on estimating the number of items that a participant can maintain over short periods of time by comparing task performance at different working memory loads (for a review see Luck and Vogel, 2013). However, more recent studies (Bays and Husain, 2008; Spencer and Hund, 2002; Zhang and Luck, 2008) suggest that increasing the detail or precision of these objects requires additional working memory resources at the cost of reducing the number of objects that can be remembered simultaneously. Hence, the formation of high-precision representations might be easier for individuals with high working memory capacity, while low working memory capacity would require a reduction of stimulus complexity, such as by using coarse categorical coding. Crawford et al. (2016) have estimated the relationship between sWM capacity and the use of fine-grained or categorical coding during sWM performance based on a sample of 778 adults. Their results showed a correlation between sWM capacity and different spatial coding strategies. A higher sWM capacity predicted higher spatial precision and lower categorical bias.

### Coding strategies relate to the engagement of separable brain systems

The assumed advantage of categorical spatial coding is that it is less demanding on cognitive resources without compromising the accuracy of responses. In contrast, encoding fine-grained information yields precise responses, but requires greater engagement of attention and cognitive control. Therefore, we hypothesized that the use of specific spatial representations would be related to the level of engagement of the attentional and control brain systems.

In the investigation of the relationship between brain activity with behavioral measures of the precision of fine-grained representations and the use of categorical representations, we observed a positive relationship between fine-grained memory precision and the delay activity in cingulo-opercular, dorsal-attention, and frontoparietal networks, and additionally, with the response activity in cingulo-opercular and dorsal-attention networks. These results suggest that increased memory precision is accompanied by increased enagagement of these networks. The cingulo-opercular, dorsal-attention, and frontoparietal networks are consistently activated in different sWM tasks and have been widely recognized to play an important role in sWM processes (Brown et al., 2004; Curtis, 2006, 2004; D’Esposito and Postle, 2015; Eriksson et al., 2015; Liu et al., 2017; Purg et al., 2022; Zarahn et al., 1999). In addition, increases in the level of activity and functional connectivity within these networks have been found to scale with increased attentional demands and working memory load (e.g., Assem et al., 2020; Barch et al., 2013; Bray et al., 2015; Cole et al., 2014; Fox et al., 2005; Liu et al., 2018; Smith et al., 2009). Several studies have provided evidence of the relationship between activity and functional connectivity in these networks and the accuracy of individual sWM performance (Assem et al., 2020; Bray et al., 2015; Liu et al., 2017; Magnuson et al., 2015). It should be noted that these studies used delayed match-to-sample tasks that do not allow separate estimation of the contribution of fine-grained and categorical representations to the observed responses. Together, these findings support the notion that the formation and active maintenance of fine-grained representations presents a cognitive load and engages attentional and cognitive control systems.

Conversely, our results revealed a positive relationship between categorical bias and delay activity in the default network, reflecting decreased inhibition in the default network related to increased use of categorical coding of spatial positions. fMRI studies investigating functional connectivity at rest have identified the role of the default network in spontaneous intrinsic activity in the absence of any cognitive load (e.g., Cole et al., 2014; Damoiseaux et al., 2006; Fox et al., 2005; Greicius et al., 2003; Moussa et al., 2012; Smith et al., 2009). Moreover, the default network shows robust deactivation during the performance of various cognitive tasks, including WM performance, which becomes stronger with increasing cognitive load (e.g., Anticevic et al., 2010; Cole et al., 2014; Fox et al., 2005; Liu et al., 2018; Raichle, 2015a,b; Smith et al., 2009). Such decreases in the activity of the default network are thought to reflect the allocation of cognitive resources to task-relevant information and protection from distraction (Liu et al., 2017). For example, Anticevic et al. (2010) have shown that stronger suppression of the default network during the encoding of target stimuli, before presentation of distractors, predicted higher response accuracy in a WM task. Therefore, these findings suggest that weaker deactivation in the default network probably reflects decreased attentional engagement and inhibition of distractors when individuals rely on categorical representations, supporting the hypothesis that categorical coding provides a less demanding sWM strategy.

Together, the observed patterns of associations between brain and behavior reflect important relationships between the two strategies of spatial information maintenance. While the positive correlation between categorical bias and pure angular error indicates a compensatory use of categorical and fine-grained representations, the two strategies relate to the engagement of separable systems. This suggests that the two strategies can be used independently and concurrently, and are enabled by different brain systems that can be used together in the service of an ongoing task. In particular, the extent of reliance on categorical representation is related to activity in the default network. The more we rely on the categorical representation, the less we deactivate the default network. However, the increased reliance on categorical representation is not directly related to changes in attentional and control networks. It may affect the extent to which we rely on the fine-grained representation, but it does not affect the quality of the fine-grained representation. The precision of the fine-grained representation is related to the level of attentional engagement, which is reflected in the activation of the attentional and control networks but not the default network.

In this study, we focused on general behavioral and neural strategies used in sWM rather than specific mechanisms. Our results provide insight into the level of general cognitive demand involved in the use of fine-grained versus categorical representations. However, they do not indicate the specific brain regions in which the different types of information are represented. fMRI studies that used multivariate pattern analysis (MVPA) have shown that fine-grained stimulus-specific information can be decoded from early sensory areas that initially processed the stimulus (Harrison and Tong, 2009; Serences et al., 2009). In contrast, other studies have shown that the prefrontal and parietal areas can store more abstract representations, such as goals, task rules, and categories (Christophel et al., 2017; D’Esposito and Postle, 2015; Meyers et al., 2008; Riggall and Postle, 2012). Based on these findings, it has been proposed that brain areas in the posterior-anterior axis respond to different levels of abstraction, with low-level posterior areas responding to fine-grained information and high-level anterior areas to more abstract information and regulatory signals (Christophel et al., 2017; D’Esposito and Postle, 2015; Rahmati et al., 2018). However, further studies are still needed to identify areas of the brain that are involved in the storage of fine-grained and categorical representations used in sWM.

### The ability to detect significant brain-behavior relationships

Several recent studies (Elliott et al., 2020; Grady et al., 2021; Marek et al., 2022; Poldrack et al., 2017) have discussed the problem of highly variable brain-behavior relationships that require large sample sizes to obtain stable and reliable results. For example, Marek et al. (2022) have shown that brain-wide association studies at typical sample sizes (i.e., around 25 participants) resulted in low statistical power, inflated effect sizes, and a failure to replicate results. We addressed this challenge by making use of a multi-site and multi-study fMRI dataset, which afforded us with a relatively large sample size (*n* = 153) compared to other task-related fMRI studies (Elliott et al., 2020; Marek et al., 2022). To the best of our knowledge, this is the largest sWM dataset to date. We also performed the analysis on *a priori* specified networks of interest in an attempt to improve the effect sizes and decrease the number of multiple comparisons. The relatively large sample size also allowed us to explore the effect of the sample size on the findings of interest.

The investigation of the effect of sample size on *β* estimates as a measure of brain-behavior relationships revealed that *β* estimates can vary substantially from sample to sample when employing relatively small sample sizes. The variability first decreases steeply and then starts to slowly approach the population mean. These findings are consistent with the observation that the sampling variability is large in small sample sizes and stabilizes at larger sample sizes (Marek et al., 2022). In the case of our study, brain-behavior associations appeared to stabilize roughly between 78 and 146 observations, consistent with the result obtained by Grady et al. (2021) and Schönbrodt and Perugini (2013). Moreover, studies have suggested very small effect sizes for univariate brain-behavior correlations (e.g. correlation coefficient *r* of 0.01) (Elliott et al., 2020; Marek et al., 2022; Poldrack et al., 2017). The mean effects sizes observed in our study ranged from small (*f*^2^ ≥ 0.02) to moderate (*f*^2^ ≥ 0.15). However, in small samples, the effect sizes varied substantially from small to high (*f*^2^ ≥ 0.35), illustrating how in small samples the effect sizes can be inflated by chance (Marek et al., 2022). Finally, similar to other fMRI studies on brain-behavior associations (Marek et al., 2022; Poldrack et al., 2017), statistical power, the ability to detect a significant effect, increased monotonically with increasing sample size, and remained fairly low even at larger sample sizes (for example, the maximum statistical power was 68.7% at *n* = 153 for the relationship between pure angular error and brain activity in the dorsal-attention network). These observations held also when using the Bayesian rather than frequentist approach to parameter estimation and statistical inference, with the Bayesian approach resulting in somewhat higher power.

In order to maximize sample size and statistical power, we combined data from multiple sites and studies, which presented additional challenges and limitations. Notably, there were minor differences in task designs and data collection protocols between studies, potentially contributing to the observed variability across participants. We addressed this issue using a multilevel approach. First, we analyzed the task condition that was directly comparable across studies and did not differ in difficulty. Second, we standardized behavioral and neural measures within each study before conducting analyses on the combined data. Third, we used a hiearachical model with study as a random effect to account for any variability due to systematic differences between studies. Nevertheless, the final sample size was still relatively small compared to the recommendation of recent studies (Elliott et al., 2020; Marek et al., 2022) indicating that thousands of participants are required to prevent the inflation of effect sizes and replication failure in brain-behavior association analyses. In addition, the hierachical structure of our data that increased the complexity of the used model might require even larger sample sizes for reliable estimates (Kerkhoff and Nussbeck, 2019; Maas and Hox, 2004, 2005). Therefore, the observed relationships may still be inflated, and more data would be needed to increase the reliability of our observations or to test their generalizability on an independent dataset.

### Conclusion

We found that during the encoding and maintenance of spatial position in WM, individuals use different types of strategies and representations. The use of these strategies appeared to be, at least to some extent, compensatory. Individuals who relied more on categorical representations showed less precision in fine-grained representations and vice versa. The use of individual strategies was reflected in the activity of separable brain networks. Increased activation of task-positive attentional and control networks was found to predict higher precision of responses, possibly reflecting the importance of attentional resources for the encoding and maintenance of fine-grained representations. In contrast, decreased deactivation of the task-negative default network was associated with increased response bias, indicating a decrease in cognitive demands when relying on categorical representations.

## Supplementary Material

Supplement 1

## Data Availability

Data and analysis scripts for this paper can be found in the Open Science Framework (OSF) repository available on https://osf.io/k8mvb/.
