# Supplementary material for: Individual differences in spatial working memory strategies differentially reflected in the engagement of control and default brain networks
Source: bioRxiv. 2023 Aug 22:2023.07.07.548112. Preprint. [Version 2] doi: 10.1101/2023.07.07.548112 (PMC10473605; doi:10.1101/2023.07.07.548112)
Supplement: Supplement 1 [file media-1.pdf]

Supplementary material: Individual differences in spatial working memory strategies differentially reflected in the engagement of control and default brain networks

Nina Purg[a*], Masih Rahmati[b], Youngsun T. Cho[b], Anka Slana Ozimič[a], Aleksij Kraljič[a], John D. Murray[b,c,d], Alan Anticevic[b,c+], and Grega Repovš[a+]

[a]Department of Psychology, University of Ljubljana, Ljubljana, Slovenia
[b]Department of Psychiatry, Yale University School of Medicine, New Haven, CT, USA
[c]Department of Psychology, Yale University School of Medicine, New Haven, CT, USA
[d]Department of Physics, Yale University, New Haven, CT, USA
[*]Corresponding author
[+]Co-senior authors

Supplementary tables

Table S1. Demographic data of participants included in the data analysis

**Study**	**Number**		**Age (years)**				**Handedness**			**Education (years)**		
	All	Females	Range	All	Females	Males	Right	Left	Both	All	Females	Males
**I**	27	18	18–38[2]	23.3 (5.8)[2]	20.9 (3.0)[2]	27.6 (7.2)	25	2	0	14.5 (2.3)[4]	13.9 (2.2)[3]	15.8 (2.1)[1]
**II**	25	16	19–31	22.8 (2.9)	22.8 (3.4)	22.9 (2.0)	23	0	2	15.2 (2.1)[2]	14.9 (2.3)[2]	15.6 (1.7)
**III**	31	22	19–42	25.0 (6.2)	22.9 (4.7)	30.1 (6.7)	30	0	1	15.2 (2.8)	14.5 (2.7)	16.9 (2.3)
**IV**	34	9	21–33	25.2 (2.8)	25.6 (3.3)	25.1 (2.7)	31	3	0	16.7 (1.6)	17.1 (1.6)	16.6 (1.6)
**V**	27	11	20–60	29.7 (9.1)	29.7 (11.5)	29.6 (7.4)	23[1]	3[1]	0[1]	16.1 (2.5)	16.2 (2.4)	16.1 (2.6)
**VI**	9	1	17–31	22.4 (4.6)	18.0 (–)	23.0 (4.6)	8	1	0	14.0 (2.1)	12.0 (–)	14.3 (2.1)
**All**	153	77	17–60[2]	25.1 (6.1)[2]	23.7 (6.1)[2]	26.5 (5.8)	140[1]	9[1]	3[1]	15.5 (2.4)[6]	15.0 (2.6)[5]	16.1 (2.1)[1]

[1] Missing information for 1 participant.
[2] Missing information for 2 participants.
[3] Missing information for 3 participants.
[4] Missing information for 4 participants.
[5] Missing information for 5 participants.
[6] Missing information for 6 participants.

Table S2. Task parameters used in different studies

Study		I	II	III	IV	V	VI
**Task**	Trials	36	32	24	20	20	80
	Blocks	2	2	3	2	1	4
**Stimuli**	Diameter (px / °va)	100 / 1.06	200 / 2.12	200 / 2.12, 2.83	125 / 1.72	125 / 1.72	125 / 1.72
	Angles (°)	5–355 (steps of 10)	7.5–352.5 (steps of 15)	7.5–352.5 (steps of 15)	9–351 (steps of 18)	9–351 (steps of 18)	9–351 (steps of 18)
	Amplitude (px / °va)	400 / 4.24	400 / 4.24	400 / 4.24, 5.66	415 / 5.72	415, 390 / 5.72, 5.38	415, 390 / 5.72, 5.38
**Events**	Fixation (s)	2.5	2.5	2.5	–	–	–
	Target (s)	0.1	2	2	1.4	1.6	1.6
	Mask (s)	0.05	–	–	–	–	–
	Delay (s)	9.85	8	8	9.8	10.4	10.4
	Attention cue (s)	–	–	–	1.4	1.6	1.6
	Response (s)	3	3	3	2.8	3.2	3.2
	ITI (s)	12.5, 15, 17.5	12.5, 15, 17.5	12.5, 15, 17.5	13.3	15.2	15.2
	ITI ratio	3:2:1	5:2:1	5:2:1	–	–	–
**Screen**	Size (mm)	640 x 400	640 x 400	640 x 400	427 x 343	427 x 343	427 x 343
	Resolution (px)	2560 x 1600	2560 x 1600	2560 x 1600, 1920 x 1200	1280 x 1024	1280 x 1024	1280 x 1024
	Viewing distance (mm)	1350	1350	1350	1385	1385	1385

Table S3. MRI parameters used in different studies

Study		I	II	III	IV	V	VI
**Scanner**		Philips Achieva 3.0T TX			Siemens 3T Tim Trio or Prisma		
**T1w and T2w**	Sagittal slices	236	236	236	224	208	208
	FOV (mm)	224 x 235	224 x 235	224 x 235	256 x 256	256 x 256	256 x 256
	Voxel size (mm)	0.7	0.7	0.7	0.8	0.8	0.8
	TR (ms)	T1: 12, T2: 2500	T1: 12, T2: 2500	T1: 12, T2: 2500	T1: 2400, T2: 3200	T1: 2400, T2: 3200	T1: 2400, T2: 3200
	TE (ms)	T1: 5.7, T2: 414	T1: 5.7, T2: 403	T1: 5.7, T2: 403	T1: 2.07, T2: 564	T1: 2.22, T2: 563	T1: 2.22, T2: 563
	Flip angle (°)	T1: 8, T2: 90	T1: 8, T2: 90	T1: 8, T2: 90	T1: 8, T2: T2 var	T1: 8, T2: T2 var	T1: 8, T2: T2 var
**BOLD**	Axial slices	48	48	48	54	72	72
	FOV (mm)	240 x 240	240 x 240	240 x 240	210 x 210	208 x 208	208 x 208
	Voxel size (mm)	3	3	3	2.5	2	2
	TR (ms)	2500	2500	2500	700	800	800
	TE (ms)	27	27	27	31	37	37
	Flip angle (°)	90	90	90	55	52	52
	SENSE factor 2	2	2	2	–	–	–
	Multi-band factor	–	–	–	6	8	8
	Number of runs	2	2	3	2	1	4
	Frames per run	215	189	281	400	770	770
**Field maps**	Axial slices	48	48	48	54	72	72
	FOV (mm)	240 x 240	240 x 240	240 x 240	210 x 210	208 x 208	208 x 208
	Voxel size (mm)	3	3	3	2.5	2	2
	TR (ms)	2500	2500	2500	731	8000	8000
	TE (ms)	27	27	27	4.92/7.38	66	66
	Flip angle (°)	90	90	90	50	90	90
**Other physiological data**		–	EEG	EEG	–	eyetracker	eyetracker

Supplementary figures

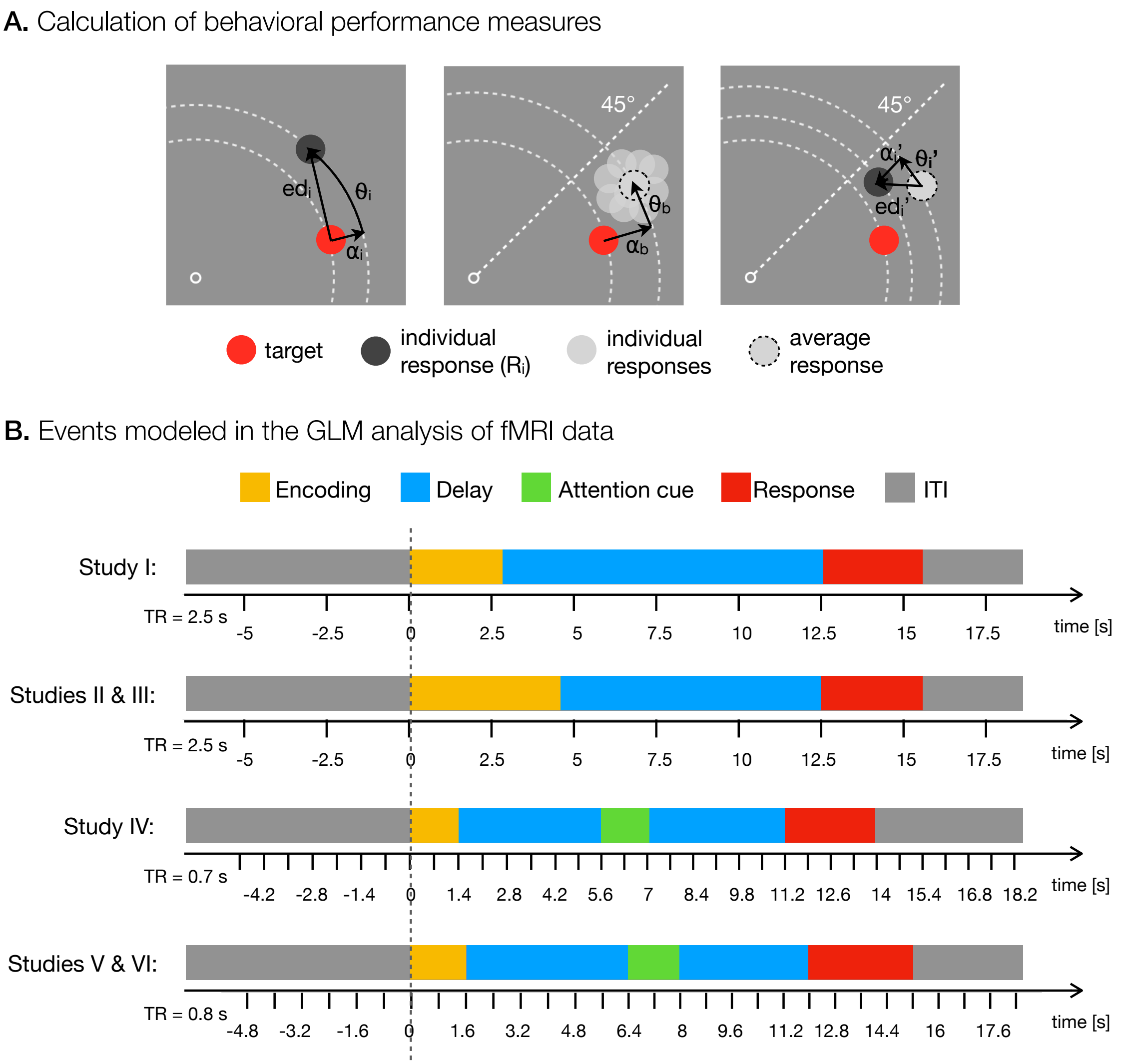

Figure S1. Visualization of behavioral and fMRI data analysis. A. Steps used in the calculation of behavioral measures of task performance; ed_i–Euclidean distance, α_i–amplitude distance, θ_i–angular distance where i denotes individual trial; α_b–amplitude bias, θ_b–angular bias. ed_i'–pure Euclidean error, α_i'–pure amplitude error, θ_i'–pure angular error; **B.** The timeline of events during a task trial modeled in the GLM analysis of fMRI data. Zeros mark the start of a task trial.

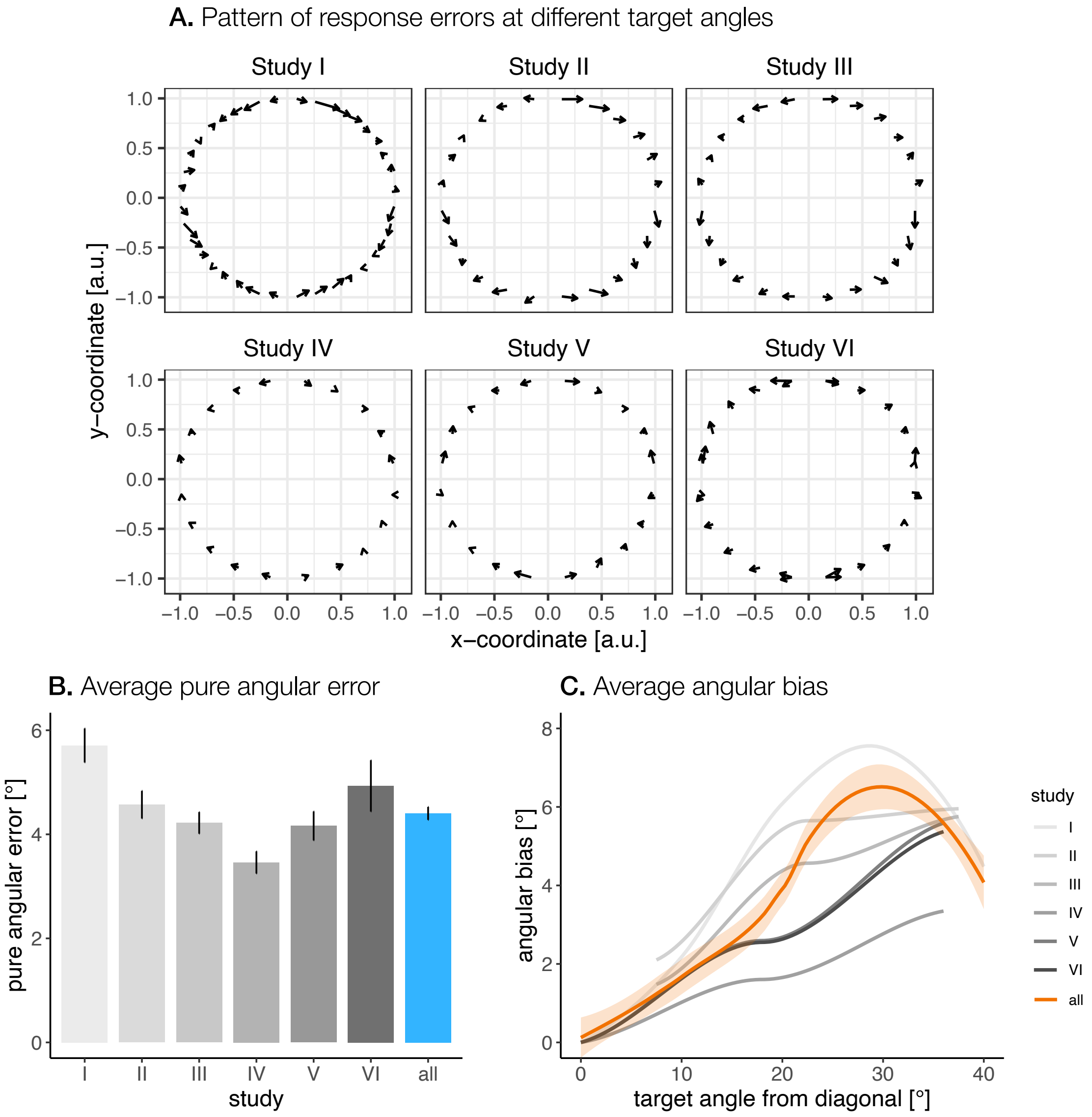

Figure S2. Systematic biases in behavioral performance. A. Pattern of average response errors at different target angles for individual studies. The start of the arrow denotes the target position, while the head of the arrow points to the average response position. **B.** Average pure angular error across all participants and for individual studies. The error bars represent the standard error. **C.** Average angular bias across all participants for individual studies. The area surrounding the average line across all participants represents standard error.

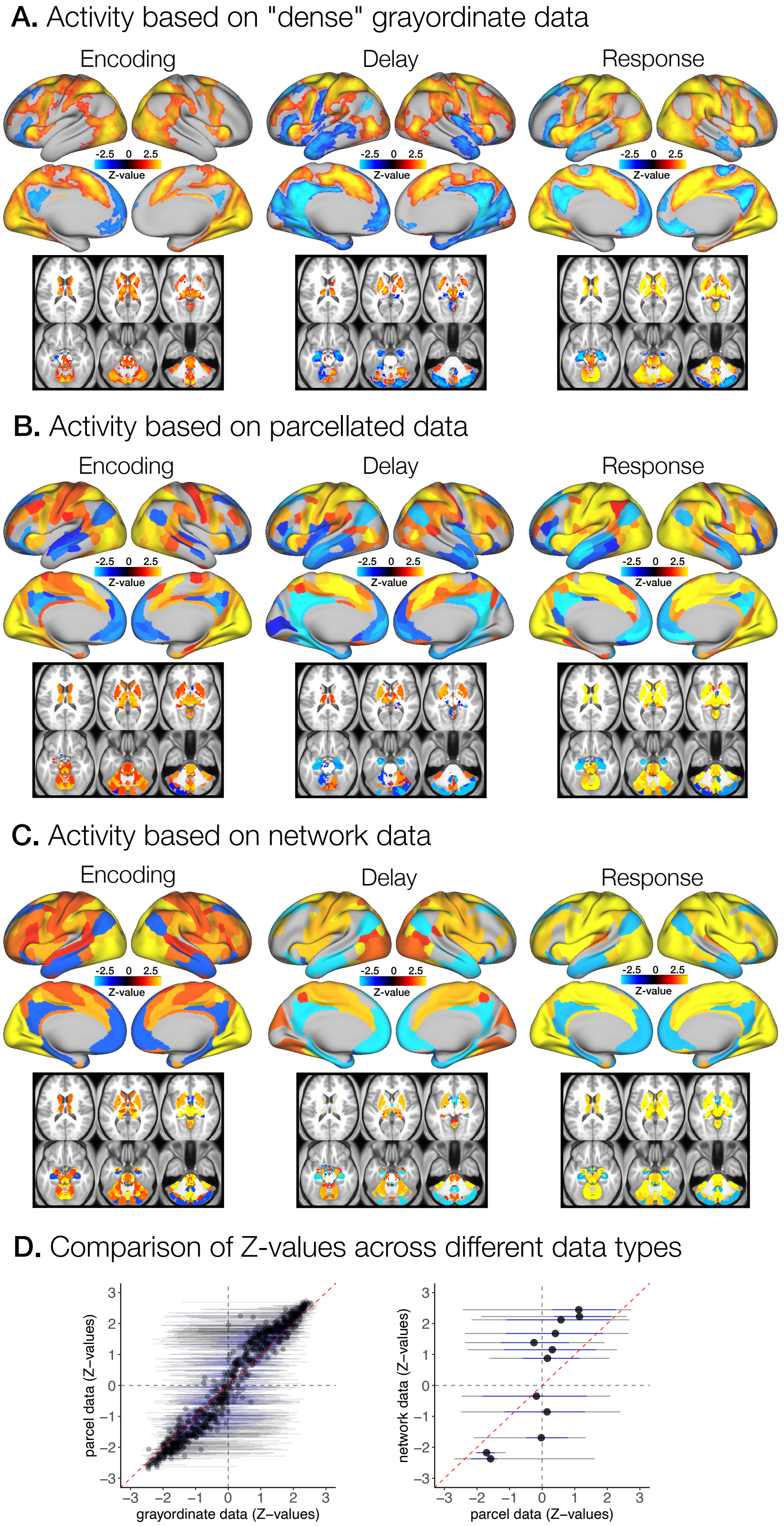

Figure S3. Brain activity during different phases of a task trial based on different levels of fMRI data parcellation. Significant activation and deactivation during the encoding, delay, and response phases for **A.** "dense" grayordinate, **B.** parcellated, and **C.** network fMRI data. p-values for "dense" grayordinate data were corrected for multiple comparisons with TFCE FWE, whereas p-values for parcellated and network fMRI were corrected with FDR. All images were thresholded at $p < 0.05$. **D.** The comparison of unthresholded Z-value maps for delay-related activity between "dense" grayordinate and parcellated data, and additionally, for parcellated and network data. The gray lines represent the range, the blue lines the inter-quartile interval (IQR), and the red dashed line the diagonal.

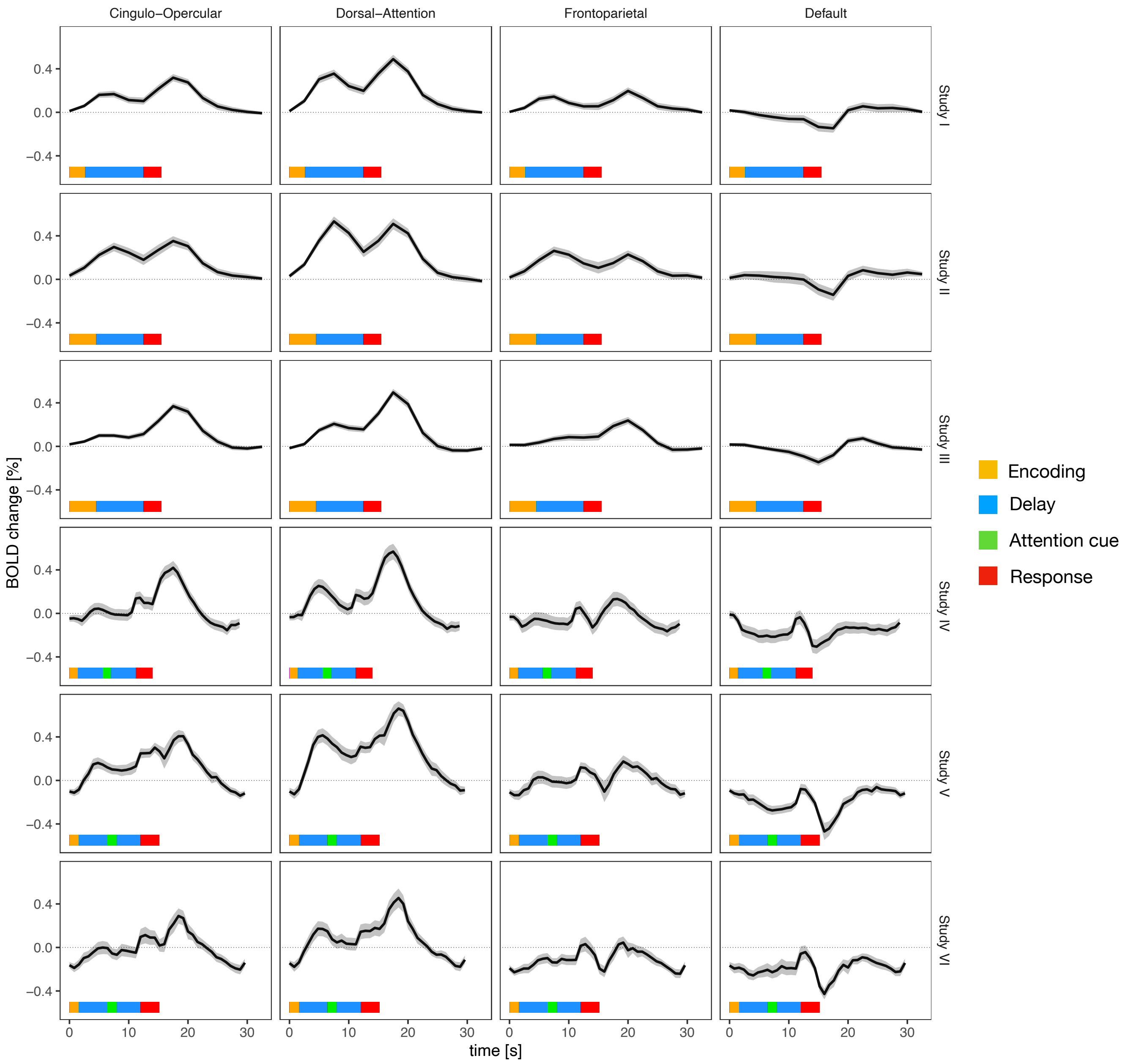

Figure S4. Average activity time series during a task trial for specific brain networks and studies. The average activity is shown for the cingulo-opercular, dorsal-attention, frontoparietal, and default networks. The shaded area represents the standard error. Colored rectangles mark the timing of different events during a task trial in different studies.

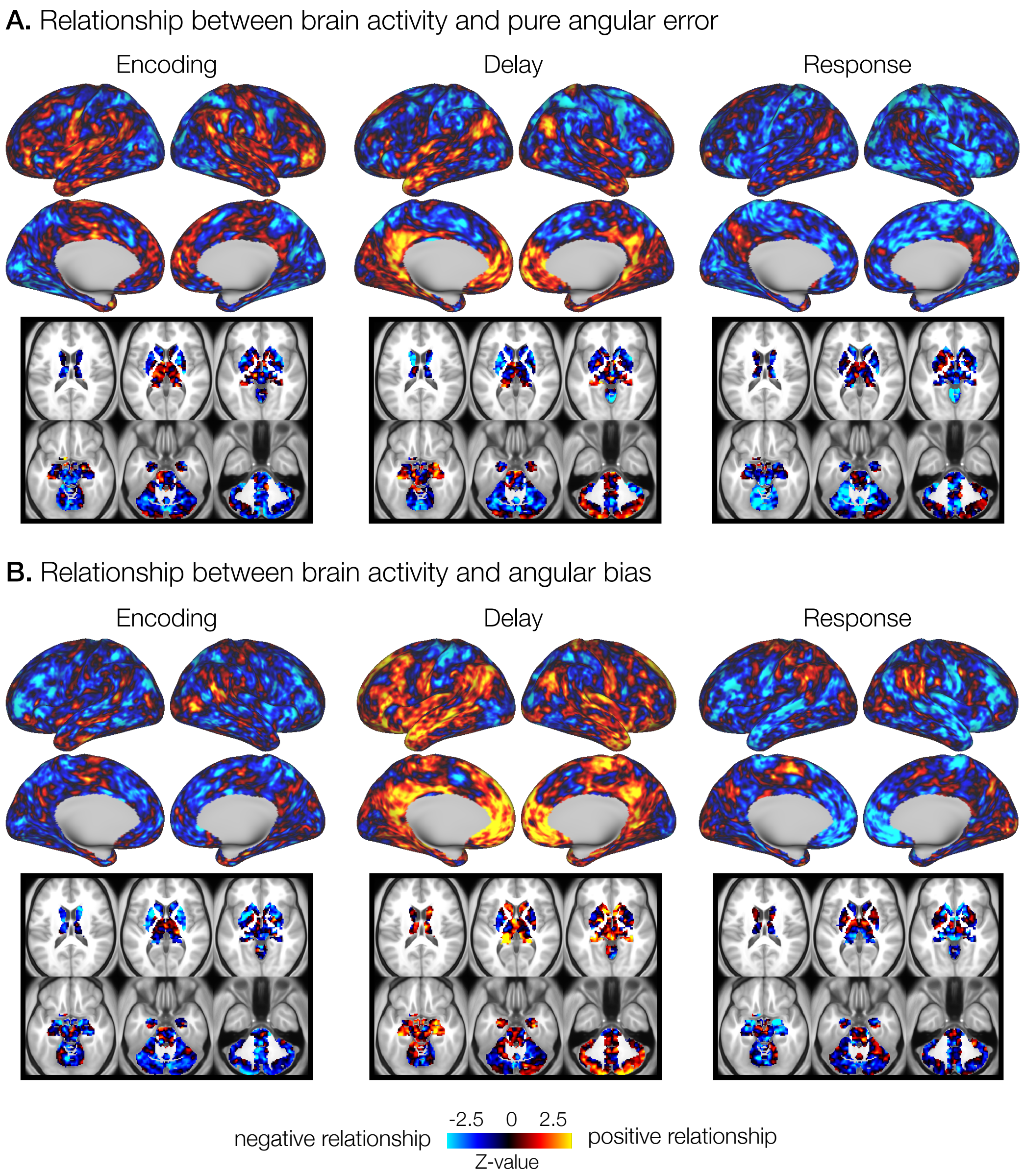

Figure S5. The relationship between brain activity based on "dense" grayordinate fMRI data and individual behavioral performance. The results are presented for the encoding, delay, and responses phases of a task trial. The relationship between activity and behavioral measures was estimated by running a linear model across participants with factors pure angular error and angular bias. The main effects of each factor for specific networks are presented.

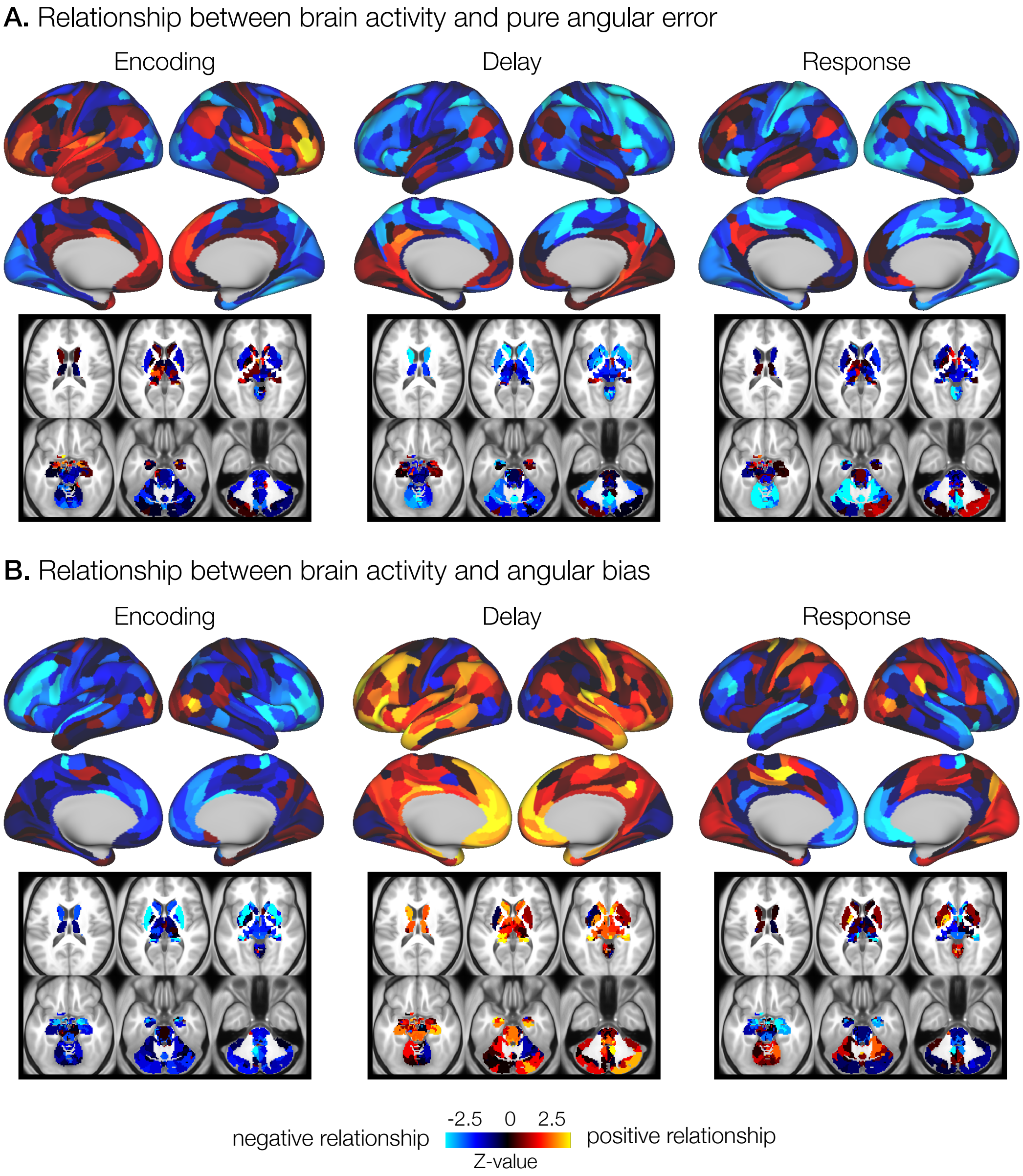

Figure S6. The relationship between brain activity based on parcellated fMRI data and individual behavioral performance. The results are presented for the encoding, delay, and responses phases of a task trial. The relationship between activity and behavioral measures was estimated by running a linear model across participants with factors pure angular error and angular bias, and study as a random effect for each task phase separately. The main effects of each factor for specific networks are presented.

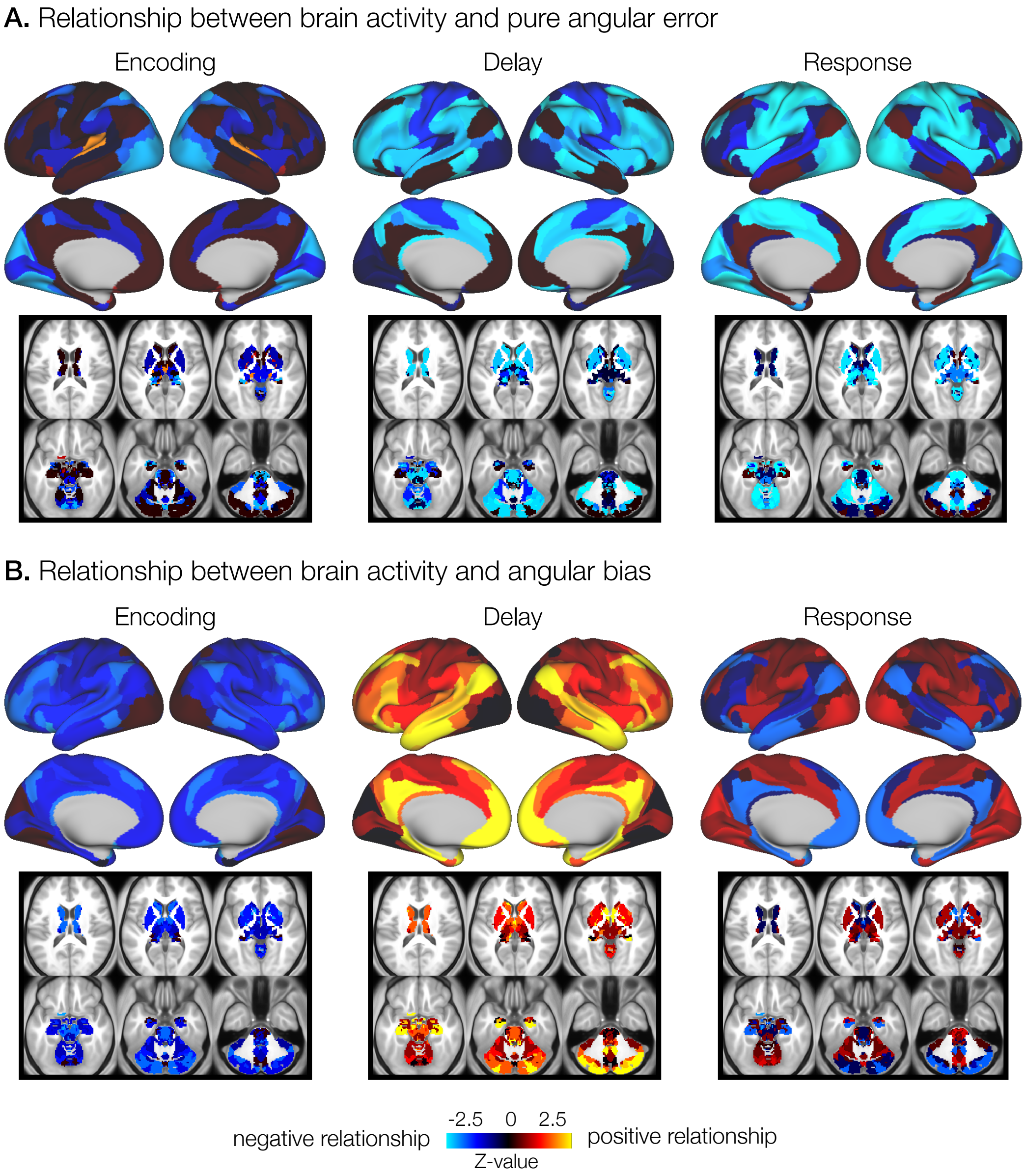

Figure S7. The relationship between brain activity based on network fMRI data and individual behavioral performance. The results are presented for the encoding, delay, and responses phases of a task trial. The relationship between activity and behavioral measures was estimated by running a linear model across participants with factors pure angular error and angular bias, and study as a random effect for each task phase separately. The main effects of each factor for specific networks are presented.

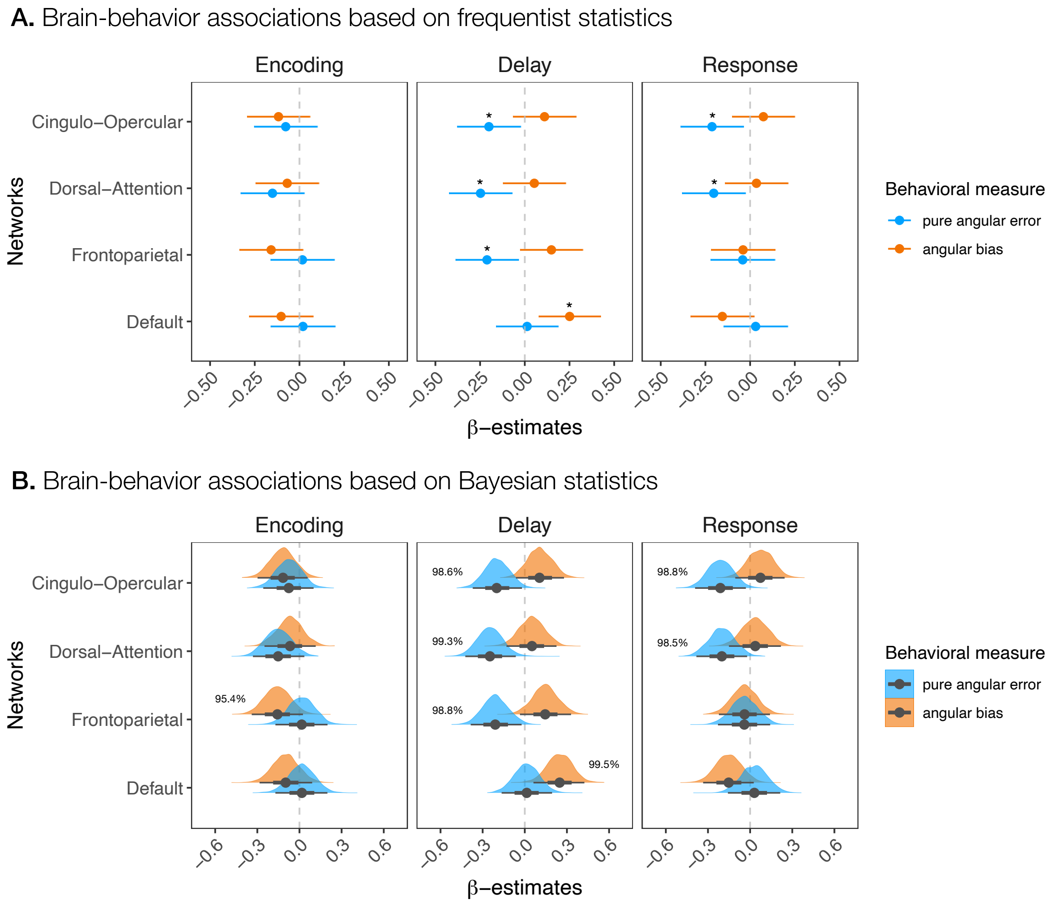

Figure S8. The relationship between the activity in selected brain networks and individual behavioral performance. The brain-behavior relationship was estimated using **A.** frequentist and **B.** Bayesian statistical approaches. **A.** Using the frequentist approach, we computed the relationship between network activity and behavioral measures by running a linear model across participants with factors pure angular error and angular bias, and study as a random effect for each network and task phase separately. The asterisks $(*)$ indicate relationships with FDR-corrected $p < 0.05$. **B.** To estimate the posterior probability of the brain-behavior relationship based on the Bayesian approach, we specified a Bayesian two-level normal linear model with factors pure angular error and angular bias. We used the study number as the grouping variable at the first level to model varying intercepts across studies. Weakly informative prior distributions were used for all model parameters. Specifically, we used normal prior distributions $(\mu = 0,\ \sigma = 10)$ for regression parameters and half-Cauchy prior distributions $(\mu = 0,\ \lambda = 2.5)$ for standard deviations. Percentages indicate the proportion of posterior distribution above or below 0.

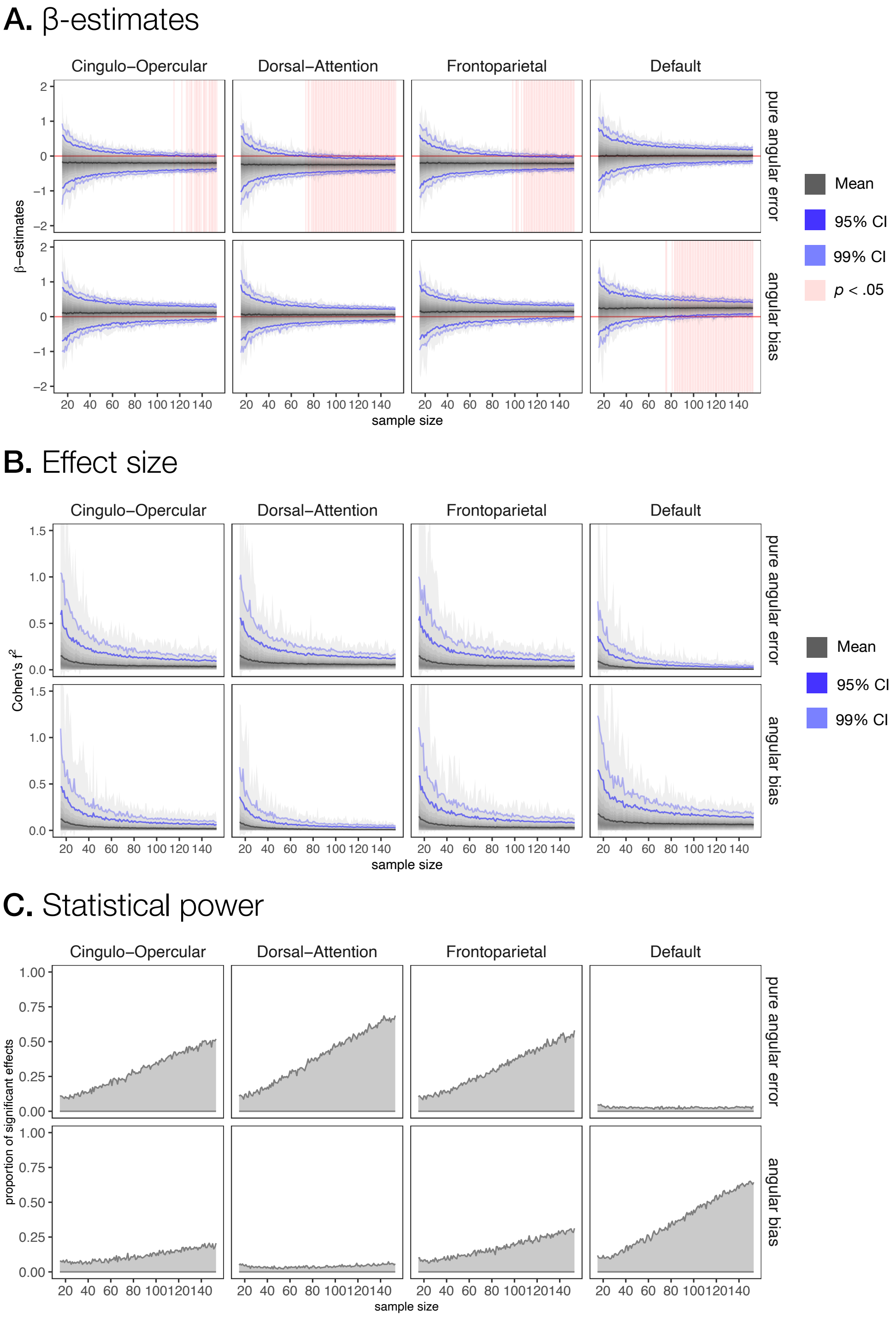

Figure S9. The effect of sample size on the ability to detect significant brain-behavior relationships based on frequentist statistics. We investigated the effect of sample size on **A.** β-estimates, **B.** effect sizes and **C.** statistical power in the investigation of the relationship of brain activity and behavioral measures of pure angular error or angular bias. Pure angular error and angular bias estimates were regressed on delay-related activity for each participant in sample sizes from 15 to 153 participants using a hierarchical model with study as a random effect variable. At each sample size, 1000 samples were created, each by sampling with replacements from the set of all participants. **A-B.** The black line denotes the mean across all samples, the grayed area denotes the span between the maximum and minimum value with darker shading reflecting the higher density, the lighter blue line denotes the upper and lower boundary for 99% of samples, the darker blue denotes the upper and lower boundary for 95% of samples. **A.** The red line denotes the value 0, the pink background shading denotes sample sizes for which the 95% confidence interval does not include 0. **C.** The proportion of samples with statistically significant effects is shown. Significance was assessed at $p < .05$ with FDR correction for multiple comparisons.

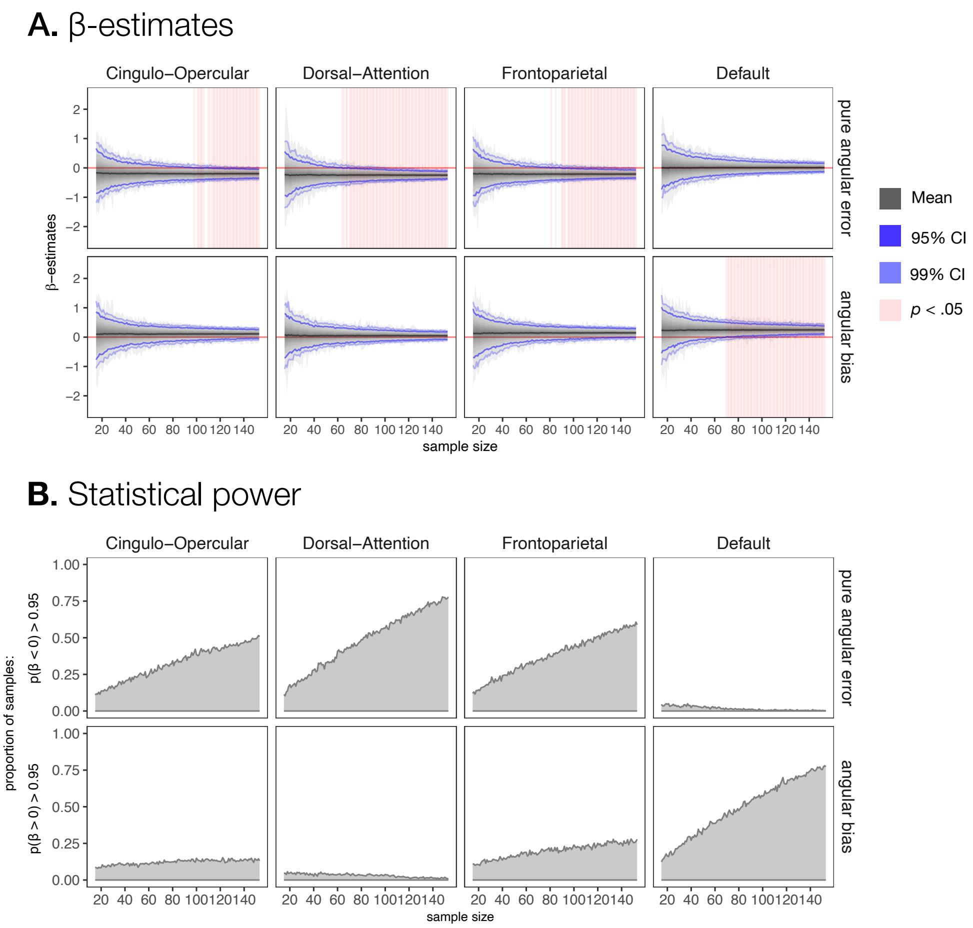

Figure S10. The effect of sample size on the estimation of brain-behavior relationships based on Bayesian statistics. We investigated the effect of sample size on **A.** β-estimates and **B.** statistical power in the investigation of the relationship of brain activity and behavioral measures of pure angular error or angular bias. The posterior probability of brain-behavior relationship was estimated using a Bayesian two-level normal linear model with factors pure angular error and angular bias, while the study number was used as the grouping variable on the first level to model varying intercepts across studies, based on a varying number of sample sizes from 15 to 153. We used normal prior distributions ($\mu = 0$, $\sigma = 10$) for regression parameters and half-Cauchy prior distributions ($\mu = 0$, $\lambda = 2.5$) for standard deviations. At each sample size, 1000 samples were created, each by sampling with replacements from the set of all participants. **A.** The black line denotes mean across all samples, the greyed area denotes the span between maximum and minimum value with the darker shading reflecting higher density, the lighter blue line denotes the upper and lower boundary for 99% of samples, the darker blue denotes the upper and lower boundary for 95% of samples. The red line denotes the value 0, the pink background shading denotes the sample sizes for which the 95% confidence interval does not include 0. **B.** The proportion of samples where 95% of posterior distribution was above or below 0.
